# Physical Activity and Dietary Composition Relate to Differences in Gut Microbial Patterns in a Multi-Ethnic Cohort—The HELIUS Study

**DOI:** 10.3390/metabo11120858

**Published:** 2021-12-09

**Authors:** Veera Houttu, Ulrika Boulund, Mary Nicolaou, Adriaan Georgius Holleboom, Aldo Grefhorst, Henrike Galenkamp, Bert-Jan van den Born, Koos Zwinderman, Max Nieuwdorp

**Affiliations:** 1 Department of Vascular Medicine, Amsterdam UMC, Location AMC at University of Amsterdam, 1105 AZ Amsterdam, The Netherlands; v.a.houttu@amsterdamumc.nl (V.H.); u.boulund@amsterdamumc.nl (U.B.); a.g.holleboom@amsterdamumc.nl (A.G.H.); a.grefhorst@amsterdamumc.nl (A.G.); b.j.vandenborn@amsterdamumc.nl (B.-J.v.d.B.); 2 Department of Experimental Vascular Medicine, Amsterdam UMC, Location AMC at University of Amsterdam, 1105 AZ Amsterdam, The Netherlands; 3 Department of Public and Occupational Health, Amsterdam UMC, Location AMC at University of Amsterdam, 1105 AZ Amsterdam, The Netherlands; m.nicolaou@amsterdamumc.nl (M.N.); heliuscoordinator@amsterdamumc.nl (H.G.); 4 Department of Clinical Epidemiology, Biostatistics and Bioinformatics, Amsterdam UMC, Location AMC at University of Amsterdam, 1105 AZ Amsterdam, The Netherlands; a.h.zwinderman@amsterdamumc.nl

**Keywords:** the HELIUS study, gut microbiota, physical activity, diet, muscle strength, calf circumference, creatinine, creatinine kinase, cross-sectional, multi-ethnic population

## Abstract

Physical activity (PA) at recommended levels contributes to the prevention of non-communicable diseases, such as atherosclerotic cardiovascular disease (asCVD) and type 2 diabetes mellitus (T2DM). Since the composition of the gut microbiota is strongly intertwined with dietary intake, the specific effect of exercise on the gut microbiota is not known. Moreover, multiple other factors, such as ethnicity, influence the composition of the gut microbiota, and this may be derived by distinct diet as well as PA patterns. Here we aim to untangle the associations between PA and the gut microbiota in a sample (*n* = 1334) from the Healthy Life In an Urban Setting (HELIUS) multi-ethnic cohort. The associations of different food groups and gut microbiota were also analyzed. PA was monitored using subjective (*n* = 1309) and objective (*n* = 162) methods, and dietary intake was assessed with ethnic-specific food frequency questionnaire (FFQ). The gut microbiota was profiled using 16S rRNA gene amplicon sequencing, and the functional composition was generated with the Phylogenetic Investigation of Communities by Reconstruction of Unobserved States (PICRUSt2). Associations were assessed using multivariable and machine learning models. In this cohort, a distinct gut microbiota composition was associated with meeting the Dutch PA norm as well as with dietary intake, e.g., grains. PA related parameters such as muscle strength and calf circumference correlated with gut microbiota diversity. Furthermore, gut microbial functionality differed between active and sedentary groups. Differential representation of ethnicities in active and sedentary groups in both monitor methods hampered the detection of ethnic-specific effects. In conclusion, both PA and dietary intake were associated with gut microbiota composition in our multi-ethnic cohort. Future studies should further elucidate the role of ethnicity and diet in this association.

## 1. Introduction

Urbanization and associated behavioral changes have led to humans being physically less active [1,2]. Insufficient physical activity (PA), defined as less than 150 min of moderate aerobic activity or 75 min of vigorous aerobic activity throughout the week, characterizes a lifestyle of approximately 28% of the adult population worldwide [2,3]. Notably, less PA contributes to a multitude of diseases, such as atherosclerotic cardiovascular disease (asCVD) and type 2 diabetes mellitus (T2DM), and hence, contributes to overall morbidity and mortality [4,5,6,7,8].

Although PA is critical in the prevention and treatment of non-communicable diseases, such as asCVD and T2DM [9,10,11], many other factors have also been shown to contribute to the development of these diseases, e.g., the composition of the gut microbiota [12,13,14,15]. Dietary habits have the greatest impact on gut microbiota composition [16,17]. Recent findings, however, indicate that exercise independently modulates the gut microbiota and gut microbial metabolism [18]. Exercise changes the composition of the gut microbiota, although the direction is still controversial since increased [19,20,21,22] as well as decreased [23,24] gut microbiota diversity has been associated with exercise. The intensity of exercise may be an important factor explaining the effect on the gut microbiota [25]. This may be partly due to the direct influence on the gastrointestinal track, i.e., gut permeability. There are also indications that the composition of the gut microbiota in athletes is associated with gut microbial metabolism and proxies of exercise, such as plasma creatinine kinase (CK) [18]. Consequently, different PA levels, moderate vs. vigorous, might also convey differential effects on the composition of the gut microbiota [13].

Thus far, most studies have focused on the extremes (professional athletes or patients with comorbidities) and there is a lack of crucial information on dietary intake and other confounders, such as age, gender and ethnicity. Different PA levels are reported in boys and girls [26], and PA preferences might change during aging [27]. Ethnicity is also of importance because ethnicity has been associated with different PA levels [28], dietary intake [29] and gut microbiota composition [30] as well as with differences in the risk for asCVD and T2DM [31,32]. A paucity of studies exist on the influence of PA on the gut microbiome on a population level and its generalizability across different populations.

Therefore, we set out to study the associations between PA, dietary intake and gut microbiota composition in a cross-sectional population-based cohort involving different ethnic groups. We used the Healthy Life In an Urban Setting (HELIUS) cohort that focusses on health and disease in population with different ethnic backgrounds in Amsterdam, The Netherlands [29,30]. We aimed to investigate whether subjectively and objectively monitored PA and/or dietary intake associate with gut microbiota composition and functionality.

## 2. Results

### 2.1. Characterization of the Study Population by Physical Activity Level

Characteristics of the included complete study population (*n* = 1334) and groups stratified by the subjective monitor (Short Questionnaire to Assess Health-enhancing physical activity (SQUASH), *n* = 1309, sedentary *n* = 441, active *n* = 868) and by the objective monitor (ActiHeart, *n* = 162, sedentary *n* = 100, active *n* = 62) are presented in Table 1. The subjective monitor data show that 66% of the study population meets the Dutch PA guideline targets. The active participants were older than the sedentary (53.4 ± 10.5 vs. 49.0 ± 10.5 years of age, t(1307) = −7.138, *p* < 0.001), had a lower body mass index (BMI) (26.7 ± 4.5 vs. 28.0 ± 5.2 kg/m^2^, t(772,552) = 4.605, *p* < 0.001) and relative fat mass (30.0 ± 9.1 vs. 31.7 ± 9.5%, t(1307) = 3.171, *p* = 0.002), as well as smaller waist circumference (93.3 ± 11.7 vs. 95.3 ± 13.6 cm, t(779,811) = 2.591, *p* = 0.007). The active participants had a higher maximum muscle strength than the sedentary participants (212.3 ± 76.6 vs. 201.0 ± 77.8 N, t(1281) = −2.493, *p* = 0.001), as well as higher creatinine levels (73 µmol/L [95% CI 72.7–76.5] vs. 75 µmol/L [95% CI 75.1–77.2], Mann–Whitney U = 208,719, *p* = 0.007). In regard to ethnicity, individuals of European descent were relatively overrepresented in the active group while other ethnicities were more frequently represented in a sedentary group, except Moroccans. Only waist-to-hip-ratio (WHR) was significantly lower in the active group compared to the sedentary group, when stratified by objective monitoring of the physical activity levels (PAL) (0.91 ± 0.1 vs. 0.93 ± 0.1, t(159) = 2.133, *p* = 0.034).

### 2.2. Dietary Intake in Relation to Physical Activity

**Figure 1:**
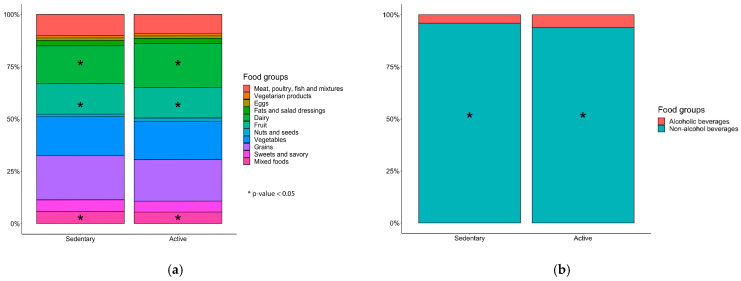
Food groups and beverages consumed by the study population stratified by the adherence to the Dutch physical activity (PA) guideline (sedentary group, *n* = 441; active group, *n* = 868) based on the Short Questionnaire to Assess Health-enhancing physical activity (SQUASH): (**a**) Stack bar of the food groups including Meat, poultry, fish and mixtures (red, organic and processed meat; chicken; fatty and lean fish; molluscs); vegetarian products (vegetarian products; meat and dairy substitutes); eggs; fats and salad dressings (margarine, butter, olive and other oils; salad dressings; red sauces; mayonnaise and similar sauces); dairy (low- and high-fat cheese and dairy products); fruit (fruits; avocado; olives); nuts and seeds; vegetables (vegetables; legumes; potatoes and tubers); grains (miscellaneous foods; low- and high fiber grains, rice and pasta); sweets and savory (savory snacks; cakes and cookies; sugar, sweets and sweet sauces; chocolate sweets); mixed foods (savory bread spreads; fast foods; soups; ethnic-specific foods); (**b**) Stack bar of the beverages including alcoholic and non-alcoholic beverages (coffee and tea; fruit juices; sugar sweetened beverages; light beverages) stratified by the activity groups (sedentary group, *n* = 441; active group, *n* = 868). Wilcoxon test was used as statistical test, *p*-values were corrected with Benjamini–Hochberg. * *p* < 0.05, active group vs. sedentary group.

The summarized data on dietary intake retrieved from the ethnic-specific semi quantitative food frequency questionnaires (FFQ) did not show significant differences between participants in the active group compared to those in the sedentary group, apart from a slightly higher daily intake of dietary fiber and alcohol consumption in the active group (Table 2). When taking total daily energy intake into consideration, the significance of the increased fiber intake by the active group disappears (2.7 ± 0.7 vs. 2.6 ± 0.7 g/MJ, t(1307) = −0.884, *p* = 0.38). When stratifying alcohol consumption by gender, we saw a significantly higher consumption in men than in women (2.9 ± 6.2 vs. 1.7 ± 3.5 g/d, t(1051,566) = 6.644, *p* < 0.001). On the other hand, when analyzing the dietary data in a more detailed manner by food groups, we saw a significantly higher consumption of fruits, mixed foods, dairy and non-alcoholic beverages in the active group compared to the sedentary group after correction for multiple testing (Figure 1A,B, Supplementary Figure S2, Wilcoxon test with Benjamini–Hochberg *p*-value adjustment).

### 2.3. Physical Activity Associates with Gut Microbiata Composition

**Figure 2:**
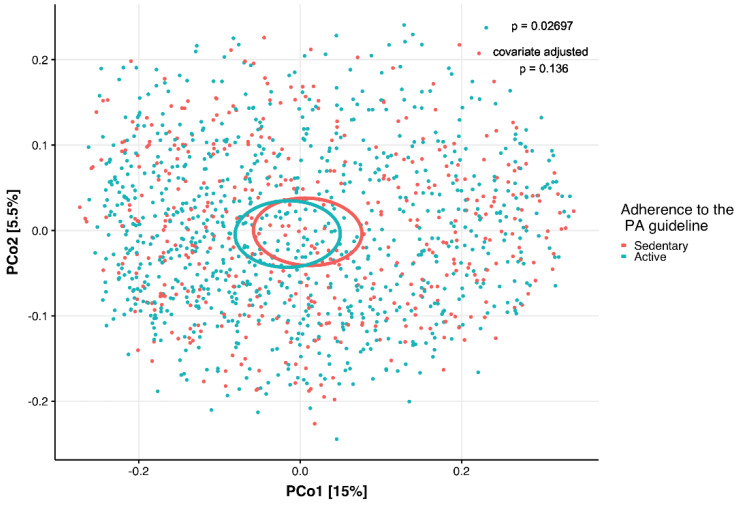
Principal Coordinate Analysis (PCoA) plot of the unweighted UniFrac distance of the study population categorized by adherence to the Dutch physical activity (PA) guideline (red dots, sedentary participants, *n* = 440, one participant with missing value for BMI was excluded); blue dots, active participants, *n* = 868; permutational analysis of variance (PERMANOVA) *p* = 0.027, covariate adjusted *p* = 0.136) based on the Short Questionnaire to Assess Health-enhancing physical activity (SQUASH). Covariates used are ethnicity, age, sex, and body mass index (BMI).

**Figure 3:**
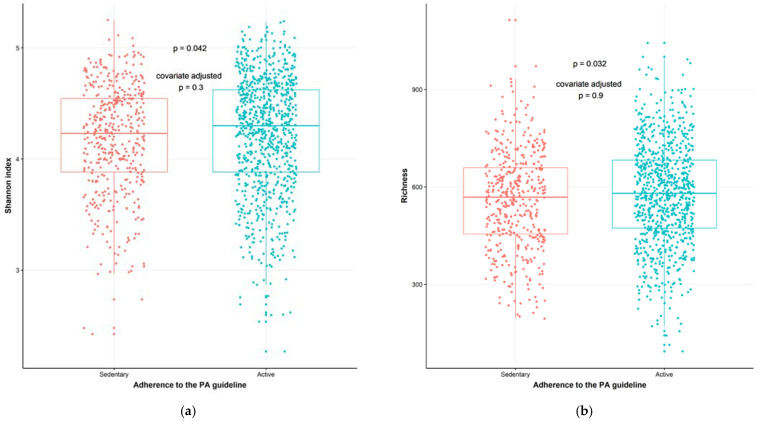
α-diversity of the gut microbiota of the study population categorized by adherence to the Dutch physical activity (PA) guideline (red dots, sedentary participants, *n* = 440 (one participant with missing value for BMI was excluded); blue dots, active participants, *n* = 868) based on the Short Questionnaire to Assess Health-enhancing physical activity (SQUASH): (**a**) Shannon index α-diversity (*p* = 0.042, covariate adjusted *p* = 0.3); (**b**) Richness (*p* = 0.032, covariate adjusted *p* = 0.9). Wilcoxon test was used as a statistical test to compare active group vs. sedentary group. Covariates used: ethnicity, age, sex and body mass index (BMI).

To study the interaction between PA, dietary intake and gut microbiota composition, we analyzed the β-diversity with a permutational analysis of variance (PERMANOVA), that indicates differences in microbial communities between individuals using both non-phylogenetic (Bray–Curtis) and phylogenetic (unweighted UniFrac and weighted UniFrac) dissimilarities. In a model adjusting for age, sex, BMI and ethnicity, the dissimilarity metrics Bray–Curtis (*p* = 0.012, R2 = 0.14%) and weighted UniFrac (*p* = 0.047, R2 = 0.17%) (which take microbial abundances into account) were significantly different between the sedentary and active groups, indicating a PA-driven association. The PA groups had different phyologenetic dissimilarity metrics (unweighted UniFrac, *p* = 0.027, R2 = 0.12%) in a model without any covariates. However, when adding the above mentioned covariates, this did not remain significant (*p* = 0.136, R2 = 0.09%) (Figure 2). In a more extensive model, adjusting for age, sex, BMI, ethnicity and diet covariates (fiber, energy and macronutrient intake), Bray–Curtis dissimilarity index (*p* = 0.022, R2 = 0.13%) and weighted UniFrac (*p* = 0.039, R2 = 0.16%) remained significant (details in Supplementary Table S1). The Shannon index diversity (*p* = 0.042, W statistic = 178,220, medians: sedentary group (4.227908), active group (4.298703), Faith’s phylogenetic diversity (*p* = 0.023, W statistic = 176,686, medians: sedentary group [30.83036], active group [31.84741]), and richness (number of species in a sample) (*p* = 0.032, W statistic = 177,526, medians: sedentary group [567.5], active group [580.5]) were higher in the active group compared to the sedentary group. These measures lost significance when adjusting for the following covariates: age, sex, BMI, and ethnicity (Figure 3A,B). The Simpson index showed the opposite, it was significant when adjusting for covariates (*p* = 0.096, W statistic = 180,626, covariate adjusted *p* = 0.002, W statistic = 171,050). Detailed analyses revealed that the relative abundance of 173 gut microbial taxa differed significantly (adjusted with covariates age, sex, BMI, ethnicity and diet) between the participants stratified by PA, indicating a PA-driven association (Supplementary Table S2). The abundance of members of Firmicutes, including *Lachnospiraceae* and *Veillonella*, were significantly higher in the active group whereas *Enterobacteriales Enterobacteriaceae, Escherichia*/*Shigella* and *Klebsiella* belonging to the phylum of Proteobacteria were more abundant in the sedentary group. Members from the phylum of Bacteroidetes, such as *Prevotella_2*, and members from the phylum of Firmicutes, such as *Roseburia hominis*, *Erysipelatoclostridium* and *Lachnoclostridium*, were also more abundant in the sedentary group.

### 2.4. The Gut Microbiome Predicts Subjectively and Objectively Monitored Physical Activity—A Machine Learning Model

**Figure 4:**
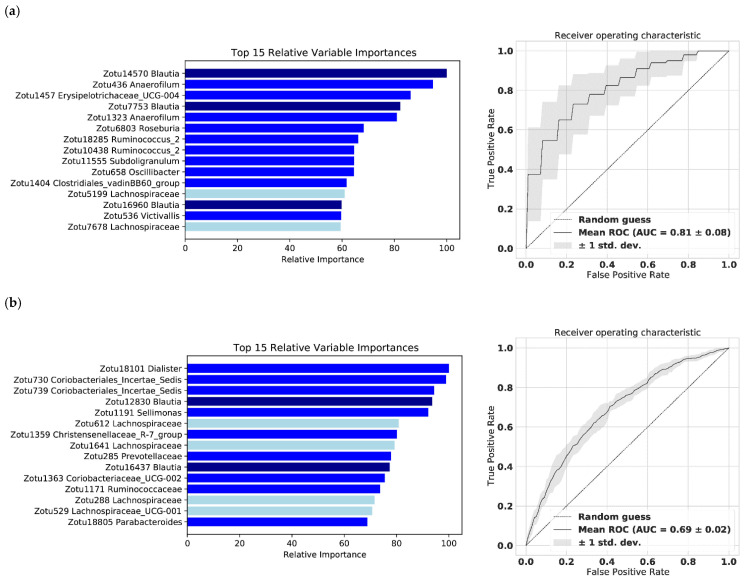
The relative variable importance for the 15 most important amplicon sequence variants (ASVs), annotated with the highest taxonomic classification and receiver operating characteristic (ROC) curve, from the machine learning of the population stratified by physical activity (PA) based on the monitoring method: (**a**) objective PA monitoring (AUC 0.81 ± 0.08, *n* = 162); (**b**) subjective PA monitoring (AUC 0.69 ± 0.02, *n* = 1309). The microbes classified as either the genus *Blautia* or the family *Lachnospiraceae* are colored with dark or light blue, respectively.

To investigate whether the gut microbiota can predict PA monitored by either subjective or objective methods, we employed a machine learning model. The model built on gut microbiota abundance was able to predict the objective monitoring (ActiHeart, *n* = 162) and the subjective monitoring (SQUASH, *n* = 1309), with an area under the curve (AUC) of 0.81 ± 0.08 and 0.69 ± 0.02, respectively (Figure 4A,B). Accordingly, gut microbiota abundance was able to predict objective monitoring without participants with antibiotic use (AUC 0.74 ± 0.08, *n* = 151) (Supplementary Figure S3). Irrespective of the type of monitoring, the most predictive bacterial taxa belong to phylum of Firmicutes, Bacteroidetes, Proteobacteria, Actinobacteria and Lentisphaerae. Specifically, *Blautia* and *Lachnospiraceae*, both members from the phylum Firmicutes belonging to the family of *Lachnospiraceae*, were predictive by both monitoring methods.

### 2.5. Parameters Related to Physical Fitness Associate with Variance of the Gut Microbiome

Since the active and the sedentary participants differed with respect to physical parameters such as calf circumference, muscle strength and creatinine (see Table 1), we next investigated whether these PA-related parameters were associated with gut microbiota composition (Table 3). As estimated by linear regression, approximately 18% of the variance in richness (*p* = 0.038) and the inverse Simpson index (*p* = 0.030) were explained by muscle strength in a model adjusted for ethnicity, age, sex, BMI and diet (energy, macronutrients and fiber). Calf circumference explained approximately 18% of the variance in richness (*p* = 0.005), 20% of the variance in Shannon index (*p* = 0.021) and 25% of the variance in Faith’s phylogenetic diversity (*p* = 0.004) in the model with ethnicity, age, BMI, sex and diet, but also in the model without dietary adjustment, indicating association independent of diet. Finally, approximately 18% of the variance in richness, 25% of variance in Faith’s phylogenetic diversity, 11% of variance in the Simpson index and 18% of variance in the inverse Simpson index were explained by creatinine in the covariate adjusted model (including covariates ethnicity, age, sex, BMI and diet). Moreover, PA-related parameters correlated (Spearman) significantly with multiple different taxa (Supplementary Table S3).

### 2.6. Functionality of the Gut Microbiome in Relation to Physical Activity and Related Parameters

**Figure 5:**
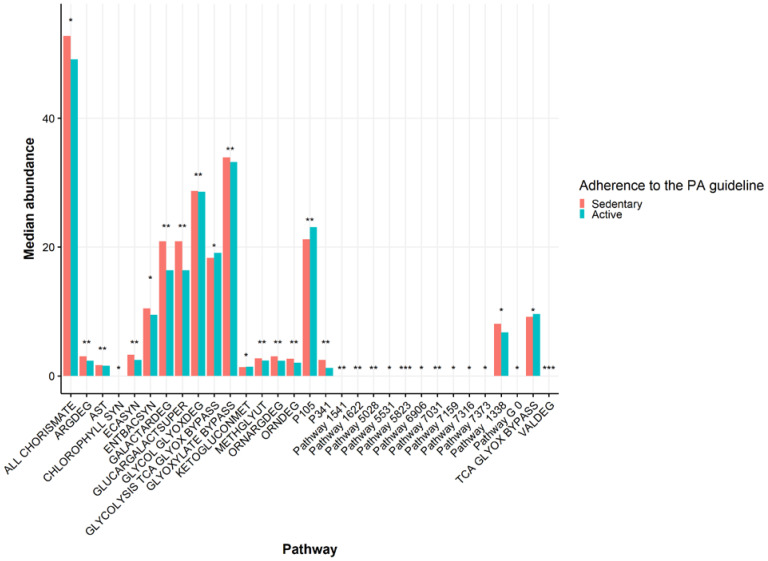
The median abundance of significantly different gut microbial pathways between the groups stratified based on the adherence to the Dutch physical activity (PA) guideline (red bars, sedentary participants, *n* = 440; blue bars, active participants, *n* = 686). Wilcoxon test was used as a statistical test. * *p* < 0.05, ** *p* < 0.01, *** *p* < 0.001, active group vs. sedentary group. Covariates used are ethnicity, age, sex and body mass index (BMI).

Due to the differences in the gut microbiota composition between the PA levels, and the PA-related parameters associated with gut microbiota composition (see Table 3, Supplementary Table S3), we analyzed taxonomically-linked metabolic pathways from the gut microbiome data by using the Phylogenetic Investigation of Communities by Reconstruction of Unobserved States (PICRUSt2) [33]. Stratified by PA levels based on SQUASH, and after adjusting for ethnicity, BMI, age and sex and upon correcting for multiple testing, 31 microbial metabolic pathways differed between the groups; when adding diet as a covariate, 23 were significant (FDR-*p* < 0.05) (Figure 5, Wilcoxon test of residuals after adjusting for covariates with linear regression. Details in Supplementary Table S4). The active group was inferred to have lower abundance of pathways related to microbial arginine metabolism (L-arginine degradation II [AST pathway], superpathway of L-arginine, putrescine, and 4-aminobutanoate degradation [ARGDEG pathway], superpathway of L-arginine and L-ornithine degradation [ORNARGDEG pathway], superpathway of L-ornithine degradation [ORNDEG pathway]). Specific taxa, such as *Enterobacteriales* and *Enterobacteriaceae*, correlated significantly with these arginine pathways (r = 0.99, FDR-*p* < 0.001), as well as *Escherichia*/*Shigella* (r = 0.86, FDR-*p* < 0.001), *Klebsiella* (r = 0.50, FDR-*p* < 0.001), Proteobacteria (r = 0.34, FDR-*p* < 0.001) and *Veillonella* (r = 0.18, FDR-*p* < 0.001). These taxa, apart from *Veillonella*, were more abundant in the sedentary group than in the active group (see Supplementary Table S1). Furthermore, the AST pathway (r = −0.10, FDR-*p* = 0.000), ARGDEG pathway (r = −0.13, FDR-*p* = 0.000), ORNARGDEG pathway (r = −0.13, FDR-*p* = 0.000), and ORNDEG pathway (r = −0.13, FDR-*p* = 0.000) correlated negatively with CK, which in turn correlated negatively with taxa such as *Enterobacteriales, Enterobacteriaceae, Escherichia*/*Shigella* and *Klebsiella* (Supplementary Table S5). Pathways related to carboxylate degradation (D-galactarate degradation I (GALACTARDEG pathway), and the superpathway of D-glucarate and D-galactarate degradation (GLUCARGALACTSUPER pathway)) were lower in the active group compared to the sedentary group. These pathways correlated positively with members of the *Bacteroides* geus (r = 0.51, FDR-*p* < 0.001). Muscle strength was another PA-related parameter, which correlated negatively with carboxylate degradation pathways (r = −0.091, FDR-*p* = 0.015).

### 2.7. Diet and Specific Food Groups Characterize the Composition of the Gut Microbiome

**Figure 6:**
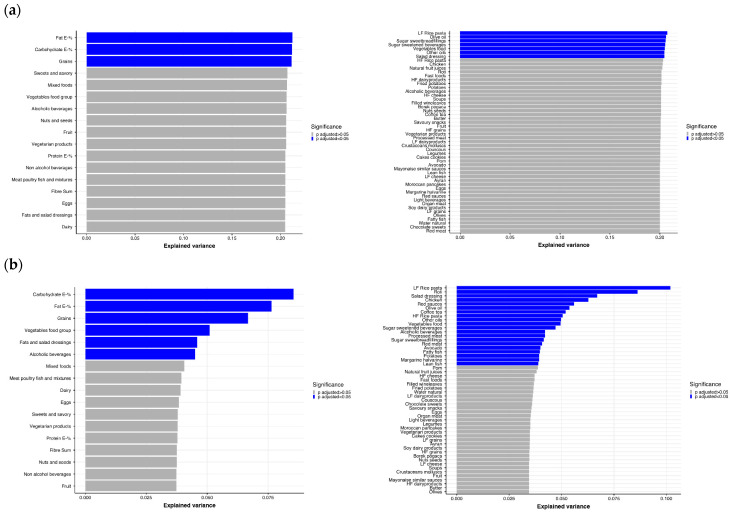
Explained variance of Shannon α-diversity index (*n* = 1308) by the major 13 foods groups (including macronutrients, carbohydrate E-%, protein E-% and fat E-%) and 52 foods in multivariable linear regression: (**a**) model adjusted for ethnicity, sex, age, and body mass index (BMI), 8 df; (**b**) model adjusted for sex, age, and BMI, 4 df. Significance level colored with blue when FDR-*p* < 0.05, grey when FDR-*p* > 0.05. E-%, energy intake expressed as energy percentage of total energy; HF cheese, high-fat cheese; HF dairy products, high-fat dairy products; HF grains, high-fiber grains; HF rice pasta, high-fiber rice pasta; LF cheese, low-fat cheese; LF grains, low-fiber grains; LF dairy products, low-fat dairy products; LF rice and pasta, low-fiber rice and pasta.

Dietary intake is one of the main drivers of gut microbiome composition [17]. Therefore, we questioned how much of the differences in gut microbiome composition were attributable to differences in dietary intake. We first explored the linear regression of parameters of dietary intake with the microbial α-diversity measured by the Shannon index. After adjusting for ethnicity, age, sex and BMI, the average intake of fat (E-%), carbohydrates (E-%) and grains had the strongest association with the Shannon index (Figure 6A). Specifically, foods including low-fiber (LF) rice and pasta, olive oil, vegetables, other oils and salad dressings within these major food groups explained a large part of the variance. Interestingly, when excluding ethnicity itself as a covariate, ethnic-specific foods, such as roti and wine-leaves explain a large part of the variance, potentially indicating how ethnic-specific foods come to act as a proxy for the effect of ethnicity on the Shannon index (Figure 6B). Of note, the largest percentage of explained variance in the Shannon index by any food, without adjusting for ethnicity, was approximately 10% (df = 4). When adding ethnicity as a covariate, the explained variance increases to approximately 20% (df = 8), suggesting that ethnicity had a rather strong effect on the analysis of food groups. Next, we investigated if specific taxa correlated with the 13 major food groups and found that different food groups show distinct correlations with different taxa (Supplementary Table S6). Vegetarian products correlated significantly with 256 taxa including species such as *Akkermansia muciniphila* (r = 0.31, FDR-*p* < 0.001). Grains correlated with 22 different taxa, while vegetables correlated (Spearman) significantly with five bacterial taxa, including *Clostridiales Lachnospiraceae* (r = 0.10, FDR-*p* = 0.01) and *Butyricicoccus* (r = 0.09, FDR-*p* = 0.03). Food groups that also showed multiple significant correlations were fruits (29 taxa); eggs (32 taxa); alcoholic beverages (89 taxa); meat, poultry and fish (30 taxa); mixed foods (28 taxa); nuts and seeds (27 taxa); sweet and savory foods (16 taxa); and salad dressings (3 taxa). The food group of non-alcoholic beverages correlated with only one taxa and dairy products did not correlate with any taxa.

## 3. Discussion

In the present study, we performed a detailed exploration on the association between fecal microbiota composition and PA, as well as dietary intake in the multi-ethnic HELIUS cohort. We found that gut microbiota composition differs between participants adhering to Dutch PA guidelines (active group) compared to the non-adhering (sedentary group). In addition to this link between PA, PA-related parameters and gut microbiota composition, the intake of specific dietary components, such as grains, was a strong factor explaining the variance in the gut microbiota composition.

We have shown that PA is associated with differences in the gut microbial diversity, and that the gut microbiota composition accurately predicts PA when measured objectively or subjectively. Yet, the direction of the associations and the most important taxa were similar in both methods. In other studies, it has been found that participants with objectively monitored high PA level have different gut microbiota diversity compared to sedentary participants [34]. The same has been described for college students whose activity was monitored by questionnaires [35], and several microbial taxa differed between active and sedentary. In our cohort, *Lachnospiraceae* was associated with PA, as well as *Blautia* belonging to the phylum of *Firmicutes*, with relative abundances being higher in active participants [36]. *Lachnospiraceae* has previously been associated with vigorous PA in the cohort of college students [35], and it has been associated together with higher richness to increased cardiorespiratory fitness (oxygen consumption [VO2]), that is likely to increase upon vigorous physical activity [37]. *Lachnospiraceae* has also been linked to the production of short-chain fatty acids (SCFA) upon exercise; the concentrations of fecal SCFA correlated positively with *Lachnospiraceae* in lean women upon an aerobic exercise program [38]. Moreover, our pairwise comparison detected *Veillonella* to be more abundant in participants adhering to the PA guideline, which is in line with recent findings that *Veillonella* is responsible for catalyzing intestinal lactate into propionate (and acetate) thereby improving the performance of athletes [21], and it also associates with vigorous physical activity in another cohort [13].

In general, higher α-diversity and richness have been associated with greater metabolic health and insulin sensitivity, which is in line with the results from our study; calf circumference associated with α-diversity and richness, and muscle strength associated with richness [39]. Improved glucose homeostasis or increased muscle endurance induced by exercise might be mediated via the gut microbiota, as seen in exercised conventional versus gut microbiota-depleted mice [40]. Muscle strength has been associated with lower risk for T2DM in the HELIUS cohort [41], and in other cohorts it was a positive predictor of survival together with calf circumference [42,43], and associated with gut microbiota composition in the elderly [44]. Beneficial metabolic effects of muscle strength can be partly due to the gut microbial changes upon exercise, but well-controlled human studies with sufficient sample size and metabolic outcomes (e.g., insulin sensitivity) are lacking. Some evidence comes from translational in vivo mice work where a fecal microbial transplant (FMT) with feces from high-functioning elderly individuals improved murine muscle strength while feces from low-functioning elderly individuals did not improve body mass and exercise endurance [45].

Consistent with previous studies, we found a positive association between plasma creatinine and microbial richness as well as α-diversity [46]. Although CK was previously associated with increased microbial diversity in rugby players, we did not find an association between gut microbiota and CK [18,22]. Interestingly, we did find an association between CK and arginine and ornithine microbial pathways. These pathways had a strong association with specific taxa such as *Enterobacteriales Enterobacteriaceae, Eschericia*/*Shigella* and *Klebsiella*, which were more abundant in the sedentary participants compared to the active participants. Hypothetically, PA might shape microbial metabolism to the needs of the host, e.g., production of energy precursors while exercising [18,21]. Yet, there is a need for more well-controlled intervention studies in different populations in order the conclude the independent effect of PA in the modulation of the gut microbiota, and its subsequent influence on metabolism.

Regarding the diet, we have shown that the intake of macronutrients was similar between sedentary and active participants, but the consumption of various food groups differed between the two groups. It has been reported that lifestyle behavior tends to cluster; health-conscious people tend to eat a healthier diet and exercise more than people with poorer lifestyle behavior, which further associates with disease prevalence [47]. Additionally, physically active individuals tend to have different dietary habits based on the type of sports, i.e., bodybuilders have higher intake of protein and lower intake of carbohydrate whereas runners show the opposite [48].

In our cohort, approximately 5% of the variance in the α-diversity Shannon index could be explained by the intake of carbohydrates, especially grains. Often, the modulatory impact of grains and carbohydrates on the gut microbiota is explained by fiber [49,50]. Fiber may influence the production of SCFA [51,52], which in turn has been associated with beneficial effects on a variety of metabolic and cardiovascular parameters, such as insulin and blood pressure [53,54]. However, in our cohort, the intake of fiber did not explain a significant part of the variance in Shannon index. Thus, the impact of foods on the Shannon diversity may not solely be driven by the fiber from grains. In contrast to the effects of fibers on the Shannon index, the intake of fat, especially oils rich in PUFA and MUFA, largely explained α-diversity as shown in another cohort [55]. Previous studies already showed that the total fat intake correlates with the Shannon index and richness where saturated fatty acids associate negatively with richness, phylogenetic diversity and number of observed taxa, while MUFA correlate negatively with number of taxa and phylogenetic diversity, and PUFA associate negatively only with phylogenetic diversity [49]. It is likely that food groups have the potential to explain the variation of the gut microbiota by capturing the synergy of different components in foods [17], such as biologically active compounds, e.g., phytochemicals and flavonoids in cereals [56,57] or chemical structures of fats [49,58]. Yet, the composition of the food groups cannot be overlooked. It is likely that some important associations were not identified since some specific foods were classified to larger groups, e.g., the food group ‘meat, poultry and fish’ combines both processed and unprocessed proteins of animal origin, though they may have distinct impacts on the gut microbiota and health [59,60]. However, the categories were kept rather wide because of the primary aim to identify associations between PA and gut microbiota. All in all, ours as well as previous studies show that dietary intake greatly explains the variation in the gut microbiota composition [14]. However, a healthy lifestyle includes both PA and healthy foods, and both are likely to influence the gut microbiota composition.

The main strength of this study is its unique multi-ethnic population, which includes participants from five different ethnic backgrounds living in the same geographical location. Another strength is the sample size of our study, the overall sample being sufficient enough, potentially one of the largest among studies investigating PA and its relationship to the gut microbiome. A large sample size is preferable when investigating gut microbiota, diseases and lifestyle [61]. However, in our cohort, the distribution of ethnicities between two PA extremes was uneven; nearly 40% of participants meeting the recommended amount of PA are of Dutch origin, influencing our analysis into the gut microbiome. We have previously shown that the ethnic background influences the composition of the gut microbiota [30]. This may be derived by distinct eating [29] as well as PA patterns [31]. However, detailed analyses comparing different ethnicities in relation to physical activity or diet were not conducted in this study due to small sample size when stratifying per ethnicity. We tried to overcome ethnic differences in dietary habits by using ethnic-specific FFQs, and by adjusting the analyses for ethnicity, but the modulatory impact of exercise on the gut microbiota is likely to be dependent on the population studied, their traditional diet and the type of exercise they perform [13].

This study has major limitations. Firstly, the cross-sectional analysis precluded derivation of causality. Secondly, self-reported PA by the SQUASH in this study is susceptible to reporting bias, and the correlation between objective and subjective PA are not as strong as previously shown [62]. Furthermore, most of the participants who wore an ActiHeart were of Dutch origin potentially influencing our analysis on the gut microbiota. Additionally, cardiorespiratory fitness is not assessed in the HELIUS cohort, and thus, we are not able to analyze this. Thirdly, 16S rRNA sequencing of the gut microbiome profiles the taxonomic composition but does not allow for a direct assessment of the functional microbial profiles. This type of sequencing is less powerful in detecting biologically relevant taxa when compared to shotgun metagenomics [63,64]. Moreover, there is no information recorded whether the stool samples were frozen or fresh when received from the study participants. This could potentially influence the results because the storage conditions of the stool samples influence the microbiota profile of the samples [65].

In conclusion, PA was associated with a distinct composition of the gut microbiome in a multiethnic population. The gut microbiota was also associated with the intake of specific dietary elements, most notably grains, independent of ethnicity. PA-related parameters such as muscle strength, calf circumference and creatinine correlated with the gut microbiota diversity. Furthermore, specific microbial pathways may be enriched in the gut microbiota of participants with different levels of PA. Together, this calls for further investigation of the influence of PA on the gut microbial composition and gut microbial metabolism, in relation to diet.

## 4. Materials and Methods

### 4.1. Study Population

The HELIUS study comprises 24,789 adult individuals (18–70 years of age) randomly sampled by ethnic origin from Amsterdam area in The Netherlands from 2011 to 2015 [32]. Participants were of Dutch, South-Asian Surinamese, African Surinamese, Ghanaian or Turkish or Moroccan origin. After a positive response, participants received a confirmation letter of the appointment for the physical examination, including a digital or paper version of the questionnaire (depending on the preference of the subject). Participants who were unable to complete the questionnaire themselves were offered assistance from a trained ethnically-matched interviewer.

This cross-sectional study was conducted with a subsample of the HELIUS cohort, namely including those of whom data on physical activity, dietary data and gut microbiota composition were present. The subsample was divided into two sets: the participants with subjective monitor data included 1309 participants (sedentary *n* = 441, active *n* = 868) who had completed SQUASH, and who had not used antibiotics in the past three months before the fecal sample (participants where this information was missing were also excluded from this set); and the participants with objective monitoring data of 162 participants (sedentary *n* = 100, active *n* = 62) who had worn an accelerometer (participants with antibiotic use (*n* = 11, unknown *n* = 14) were included in this dataset).

### 4.2. Body Composition, Function, and Biochemistry

Anthropometrics, including weight, height, BMI, waist and hip circumference and WHR, as well as calf and thigh circumferences were assessed in the study visit. Body composition was assessed by arm-to-leg bioelectrical impedance analysis (BIA) that measures impedance, resistance, and reactance in Ohm at 50 Hz (Bodystat 1500 analyzer, Bodystat Ltd., Isle of Man, Cronkbourne, Douglas, UK). Plasma creatinine and CK levels were determined from fasting venous blood samples using standard laboratory techniques. Muscle strength was measured as handgrip strength with the Citec handheld dynamometer (CIT Technics, Haren, The Netherlands). The average of the two highest measurements in Newton (N) from both hands within one minute intervals was used for the final value as previously described [41].

### 4.3. Physical Activity

#### 4.3.1. Subjective Physical Activity Monitor

PA was monitored by the SQUASH, which was developed by the Dutch National Institute of Public Health and the Environment (RIVM) [66]. It assesses self-reported daily activities, including commuting in leisure, household and occupation time (i.e., walking and cycling) as well as other exercise habits (i.e., gardening and swimming); it also indicates whether PA was in accordance with the Dutch PA guideline. Self-reported activities per day were converted to minutes per week (min/week). If weekly PA was more than 30 min per session and it was carried out at least five days per week (in total of 150 min/week), it was considered to meet the Dutch PA guidelines, which is in accordance with the international PA guideline of the World Health Organization (WHO) for a general population [3,67].

#### 4.3.2. Objective Physical Activity Monitor

Participants in a subsample of the population (*n* = 162) wore a validated accelerometer with electrocardiography (ECG) electrodes (ActiHeart, CamNtech Ltd., Papworth, UK) [68] to objectively monitor PAL for four consecutive days. In this study, PA is considered sedentary when PAL is below or equal 1.69 and active when PAL is above or equal 1.70.

### 4.4. Dietary Intake and Food Groups

Information on dietary intake was derived from FFQs [69] Specifically, the daily average intake of energy (kilocalories per day (kcal/d)), macronutrients and fatty acids (energy percentages (E-%)) and dietary fiber (grams per day [g/d]; fiber per energy intake (g/MJ) was calculated by transforming kcal to MJ and dividing fiber intake with MJ) were retrieved. Additionally, the FFQ included approximately 200 food items classified into 52 food groups based on similarity in nutrient profile or culinary according to the Dutch food composition database (NEVO) constructed by the RIVM. These 52 foods were further classified into 13 different food groups (Supplementary Figure S1).

### 4.5. Fecal Gut Microbiome Composition and Functionality

Stool samples were received by members of the study staff in the morning of a physical examination within six hours after the collection, or the next morning after the physical examination [30]. In the latter case, participants were asked to store the stool sample in their freezer until bringing it to the research location. There is no information available whether samples were received fresh or frozen. Stool samples were transported daily to −80 degree freezers at the Academic Medical Center (AMC) for storage from a temporary storage of −20 degrees at the Academic Medical Center (AMC) for storage from a temporary storage of −20 degrees at the research location.

#### 4.5.1. Profiling of Fecal Microbiota Composition

Library preparation and sequencing of the gut microbiota was performed at the Wallenberg Laboratory (Sahlgrenska Academy, the University of Gothenburg, Sweden). For this, total genomic DNA was extracted from a 150 mg fecal sample aliquot using a repeated bead beating method as previously described [30]. In order to profile the composition of fecal microbiota, the V4 region of the 16S rRNA gene were sequenced on a MiSeq system (RTA v. 1.17.28, bundled with MCS v. 2.5; Illumina, San Diego, CA, USA) with 515F and 806R primers (2 × 250 bp paired-end reads). Amplification of 16S rRNA genes were done in duplicate reactions with a reaction mixture containing 1 × Five Prime Hot Master Mix (5PRIME GmbH), total of 400 nM of reverse and forward primers, 0.4 mg/mL bovine serum albumin (BSA), 5% dimethylsulfoxide, and 20 ng of genomic DNA (total volume of 25 μL). PCR steps were run as presented in Table 4. Combined duplicates were purified (NucleoSpin Gel, PCR Clean-Up kit, Macherey-Nagel, Düren, Germany), and quantified (Quant-iT PicoGreen dsDNA kit, Invitrogen, Waltham, MA, USA). Purified PCR products were diluted to 10 ng/μL and pooled in equal amounts, and to remove short amplicons, those were purified again (Ampure magnetic purification beads, Agencourt, Beverly, MA, USA). The absence of detectable PCR products in negative controls was confirmed with gel electrophoresis. The protocol used to analyze the samples was optimized using mock samples; and thus, there were no positive controls. Libraries for sequencing were prepared by mixing the pooled amplicons with PhiX control DNA (Illumina) resulting in a concentration of 3 pM input DNA (15% PhiX). It generated around 700 K clusters/mm^2^. Quality score was over 30 in 70% of the bases. Analytical procedures were blinded (non-randomized) for ethnicity.

#### 4.5.2. Processing of 16s rRNA Gene Reads and ASV Generation

USEARCH (v11.0.667_i86linux64) was used to analyze the raw sequencing reads [70]. For the paired-end merging, the following parameters were used: fast_mergepairs and maxdiffs = 30, fastq_filter and fastq_maxee = 1. After the merging and quality filtering, contigs were dereplicated and unique sequences were denoised using UNOISE3, to obtain amplicon sequence variants (ASVs). Then all merged reads were mapped to the resulting ASVs to generate the ASV table. The ASVs that did not match the expected amplicon length (ASVs longer than 260 base pair or shorter than 250 base pair) were filtered out. The ‘assign Taxonomy’ function from the dada2 R package (v 1.12.1) and the SILVA (v. 132) reference database were used to assign taxonomy [71,72]. MAFFT (v. 7.427) with default settings was used to align ASVs [73,74]. The ‘double precision’ build of FastTree (v. 2.1.11) was used to build a phylogenetic tree based on the multiple sequence alignment, with a generalized time-reversible model (‘-gtr’) [75]. These components (phylogenetic tree, taxonomy and ASV table) were integrated with the ‘phyloseq’ R package (v. 1.28.0) [76]. The ‘vegan’ R package (v 2.5-6) was used to rarefy the ASV table was rarefied to 14,932 counts per sample. 24 of the 6056 sequenced samples had <5000 counts per sample and were excluded at the rarefaction stage. The final dataset contained 6032 samples and 22,532 ASVs. The functional composition data from the 16s sequencing data were generated using Phylogenetic Investigation of Communities by PICRUSt2 (v. 2.3.0-b) [33], and specific pathways and their classification were identified using the MetaCyc database.

#### 4.5.3. Characteristics of Gut Microbiota Composition

The dissimilarities in gut microbiota composition between individuals (β-diversity) were assessed with the Bray–Curtis dissimilarity index, as well as weighted and unweighted UniFrac distance calculated at the ASV level (function ‘vegdist’ vegan v. 2.5-6 R package for Bray–Curtis and function ‘UniFrac’ phyloseq v 1.30.0 R package for weighted and unweighted UniFrac [77]. Unconstrained Principal Coordinate Analysis was used to plot the Bray–Curtis dissimilarities (function pcoa ape v. 5.4-1 R package). The α-diversity of gut microbiota for each individual was assessed with several indices calculated at the ASV level: richness (number of unique ASVs: function ‘estimate’ vegan R package), the Shannon index, the Simpson index, Inverse Simpson index, the inverse Simpson index (function diversity ‘vegan’ R package) and Faith’s phylogenetic diversity (function pd ‘picante’ v. 1.8.2 R package).

#### 4.5.4. Microbial Data Preparation

The microbial data were summarized to phylum, family, genus, species and ASV level, this was then filtered to only keep taxa with >20 counts in >5% of participants. For the machine learning analyses, an unfiltered ASV table for either 1309 participants or 162 participants was used as input data.

### 4.6. Statistical and Bioinformatics Analysis

#### 4.6.1. Statistical Analysis of Clinical Outcomes

Conventional statistical analyses were performed with IBM SPSS Statistics Software Version 26.0 (IBM Corp., Armonk, NY, USA). Distribution of the parametric variables was checked with the homogeneity test of variances and by inspecting visually the Q-plots and histograms. Normally distributed continuous variables were analyzed using Fisher *T*-tests, and categorized variables were analyzed using chi square test. Skewed distributed variables were tested with a Mann–Whitney test. Pearson correlation was used for correlation of PA levels from the different PA monitor methods (objective and subjective), and Pearson chi square was used for correlation between the categories (active and sedentary). The statistical significance level was set to *p* < 0.05. An unconstrained principal component analysis of the food groups of the set of 1309 participants was created, after removing missing values (using function prcomp ‘stats’ v. 3.6.3 R package, with scale = TRUE in R).

#### 4.6.2. Statistical Analysis of Gut Microbiota Composition

Β-diversity was tested with a PERMANOVA (‘adonis’, permutations = 1000, in R). Analyses were adjusted for the following covariates: age, sex, BMI, and ethnicity, and if applicable, where noted for dietary intake (fat (E-%), protein (E-%), carbohydrate (E-%), fiber (g/d) and total daily energy intake (kcal/d)). For the small set of 162 participants (where PAL outcomes were used; sedentary PAL < 1.69, active PAL > 1.70), antibiotic use in the past three months was also included as a covariate. Residuals of taxa, pathway and gene abundances as well as α-diversity indices were computed using ‘lm’ in R, adjusting for the above mentioned covariates, which were compared between binary outcomes with a Wilcoxon test. Spearman correlation tests were performed on the covariate adjusted residuals between taxa abundances and continuous outcomes. In all analyses, an adjusted *p*-value (p.adjust with Benjamini–Hochberg [78] method in R) < 0.05 was considered significant. The explained variance of the Shannon index α-diversity by the different outcomes (foods, food groups and macronutrients) was calculated using ‘lm’ in R, adding covariates for age, sex, BMI and where noted, ethnicity.

#### 4.6.3. Machine Learning

To predict the binary outcomes (objective monitoring method with two PAL levels (ActiHeart) and subjective monitoring method (SQUASH) with two activity levels stratified based on adherence to the PA guideline), the input features were the microbial data (see above section on Microbial Data Preparation). The input variables (ASV abundances) were preprocessed by an initial filtering on minimum occurrence ratio (the taxa must be present (>1 count) in >5% of participants), and a univariate feature selection (SelectPercentile with f_classif). Thereafter the data was split into an 80% training set and 20% testing set, which was randomly shuffled using Stratified Shuffle Split over 10 iterations to ensure stable predictions, and the average AUC of all iterations was reported. In each shuffle, XGBoost XGBregressor (objective = ‘reg:squarederror’) was used to predict binary outcomes (SQUASH and PAL activity levels) with GridSearch using roc_auc as the scoring function. A random variable was appended in each shuffle to serve as a threshold for relevant features selected by the model. Internal five-fold cross validation was performed in the hyperparameter search. The parameter grid used was: ‘max_depth’: (3–5), ‘learning_rate’: (0.01, 0.1, 0.2), ‘n_estimators’: (200), ‘min_child_weight’: (1, 2), ‘gamma’: (0, 0.1, 0.2), ‘subsample’: (0.5, 0.8, 1), ‘colsample_bytree’: (0.5, 0.8, 1).

## Figures and Tables

**Table 1:** Characterization of the study population by the adherence to the Dutch physical activity (PA) guideline based on the Short Questionnaire to Assess Health-enhancing physical activity (SQUASH), and by PA level based on ActiHeart. Chi-Square for categorical variables, t-test for parametric variables and non-parametric Mann–Whitney test were used for statistical testing to compare the active vs. sedentary group. Data are expressed as means ± SD, absolute numbers with percentages or medians with 95% CI.

	Complete	SQUASH: Subjective Monitoring				ActiHeart: Objective Monitoring			
		Complete	Sedentary	Active	*p*-Value	Complete	Sedentary	Active	*p*-Value
*n*	1334	1309	441	868		162	100	62	
Age	51.0 ± 10.8	51.9 ± 10.7	49.0 ± 10.5	53.4 ± 10.5	<0.001	51.1 ± 7.4	51.8 ± 6.5	50.0 ± 8.5	0.125
Seks (*n*, %)									
Men	647 (48.5)	633 (48.4)	203 (46.0)	431 (49.1)	0.187	76 (46.9)	47 (47.0)	29 (46.8)	0.975
Women	687 (51.5)	673 (51.4)	238 (54.0)	435 (50.9)	0.187	86 (53.1)	53 (53.0)	33 (53.2)	0.975
Ethnicity (*n*, %)									
AS	170 (12.7)	166 (12.7)	75 (17.0)	91 (10.5)	0.001	13 (8.0)	6 (6.0)	7 (11.3)	0.250
SAS	99 (7.4)	92 (7.0)	46 (10.4)	46 (5.3)	0.001	11 (6.8)	5 (5.0)	6 (9.7)	0.228
Maroccan	346 (25.9)	344 (26.3)	110 (24.9)	234 (27.0)	0.434	43 (26.5)	28 (28.0)	15 (24.2)	0.597
Turkish	286 (21.4)	281 (21.5)	109 (24.7)	172 (19.8)	0.041	32 (19.8)	22 (22.0)	10 (6.2)	0.362
Dutch origin	434 (32.5)	426 (32.5)	101 (22.9)	325 (37.4)	<0.001	63 (38.9)	39 (39.0)	24 (38.7)	0.971
Weight (kg)	77.4 ± 15.0	77.4 ± 14.9	79.4 ± 16.5	76.4 ± 14.0	0.001	76.7 ± 14.9	76.8 ± 15.0	76.7 ± 14.9	0.971
BMI (kg/m^2^)	27.1 ± 4.8	27.3 ± 4.8	28.0 ± 5.2	26.7 ± 4.5	<0.001	26.5 ± 4.6	26.6 ± 4.7	26.2 ± 4.4	0.577
Fat mass (%)	30.6 ± 9.2	30.6 ± 9.3	31.7 ± 9.5	30.0 ± 9.1	0.002	30.9 ± 7.8	31.4 ± 7.9	30.1 ± 7.6	0.314
WC (cm)	94.0 ± 12.4	94.0 ± 12.4	95.3 ± 13.6	93.3 ± 11.7	0.007	93.1 ± 11.5	93.9 ± 10.8	91.7 ± 12.5	0.235
WHR	0.92 ± 0.1	1.00 ± 2.1	0.91 ± 0.1	0.98 ± 1.7	0.292	0.92 ± 0.1	0.93 ± 0.1	0.91 ± 0.1	**0.034**
TC (cm)	58.8 ± 10.3	58.9 ± 10.4	59.8 ± 10.2	58.4 ± 10.4	0.022	58.2 ± 9.8	58.0 ± 8.4	58.6 ± 11.8	0.733
CC (cm)	37.5 ± 3.4	37.5 ± 3.4	37.9 ± 3.9	37.3 ± 3.2	0.001	37.6 ± 3.4	37.9 ± 3.6	37.2 ± 3.1	0.233
Muscle strength (N)	208.6 ± 76.9	208.5 ± 76.9	201.0 ± 77.8	212.3 ± 76.2	0.013	209.5 ± 69.1	208.0 ± 70.0	211.7 ± 68.3	0.745
Creatinine (µmol/L)	74 (74.6–76.5)	74 (74.7–76.6)	73 (72.7–76.5)	75 (75.1–77.2)	0.007 *	74.0 (72.6–77.4)	76.8 (71.1–76.7)	72.5 (72.4–81.2)	0.642 *
CK (µmol/L)	119 (146.7–161.9)	120 (147.2–162.7)	112 (141.1–175.7)	123 (145.5–160.9)	0.141 *	123.5 (132.8–167.0)	117.0 (122.0–159.6)	134.0 (131.2–197.9)	0.305 *

AS, African Surinamese; BMI, body mass index; CC, calf circumference; CK, creatinine kinase; N, newton; SAS, South-Asian Surinamese; TC, thigh circumference; WC, waist circumference; WHR, waist-to-hip-ratio. * Non-parametric Mann Whitney U test.

**Table 2:** Total daily dietary intake of macronutrients and energy, and daily alcohol consumption and fiber intake in the study population stratified by the adherence to the Dutch physical activity (PA) guideline. Data are expressed as means ± SD. *T*-test was used for statistical testing to compare the active group vs. sedentary group.

	Overall (*n* = 1309)	Sedentary (*n* = 441)	Active (*n* = 868)	*p*-Value
Energy (kcal/d)	2280.5 ± 975.3	2211.9 ± 926.0	2317.2 ± 996.2	0.056
Carbohydrates (E-%)	45.0 ± 9.4	45.2 ± 7.8	44.7 ± 8.3	0.414
Protein (E-%)	16.4 ± 4.7	16.5 ± 3.1	16.2 ± 3.3	0.917
Fat (E-%)	32.0 ± 8.5	31.3 ± 6.3	31.7 ± 6.5	0.912
SFA (E-%)	11.0 ± 6.4	10.8 ± 2.9	10.8 ± 3.2	0.503
MUFA (E-%)	12.6 ± 4.3	12.6 ± 3.2	12.5 ± 3.3	0.925
PUFA (E-%)	7.8 ± 4.0	7.4 ± 2.3	7.7 ± 2.3	0.343
Alcohol (g/d)	7.1 ± 13.0	5.0 ± 11.1	8.1 ± 13.8	<0.001
Fiber (g/d)	24.7 ± 10.7	23.5 ± 9.6	25.1 ± 10.7	0.006

E-%, energy intake expressed as energy percentage of total energy; g/d, grams per day; kcal/d, kilocalories per day; MUFA, monounsaturated fatty acids; PUFA, polyunsaturated fatty acids; SFA, saturated fatty acids.

**Table 3:** The associations between parameters related to physical activity (PA) (muscle strength, calf circumference (CC), thigh circumference (TC), creatinine and creatinine kinase (CK)) and gut microbiota composition (richness, α-diversity Shannon index, Faith’s phylogenetic diversity, Simpson index and inverse Simpson index) by multivariable linear regression models. Model 1 adjusted for ethnicity, sex, age, body mass index (BMI), 8 degrees of freedom (df); Model 2 = Model 1 plus diet (energy, macronutrients, and fiber), 13 df. N = 1308.

			Muscle Strength	CC	TC	Creatinine	CK
Richness	Model 1	R2	0.251	0.171	0.166	0.171	0.169
		R2 adj.	0.244	0.166	0.160	0.166	0.164
		β	0.121	5.322	0.282	0.679	0.046
		*p*-value	0.119	0.005	0.525	0.016	0.128
	Model 2	R2	0.185	0.186	0.181	0.185	0.183
		R2 adj.	0.178	0.179	0.174	0.178	0.176
		β	0.154	5.106	0.216	0.682	0.040
		*p*-value	0.038	0.005	0.609	0.012	0.174
Shannon index	Model 1	R2	0.207	0.209	0.207	0.213	0.205
		R2 adj.	0.202	0.204	0.202	0.208	0.200
		β	0.000	0.016	0.003	0.003	0.000
		*p*-value	0.087	0.010	0.044	0.000	0.504
	Model 2	R2	0.227	0.227	0.225	0.002	0.224
		R2 adj.	0.220	0.220	0.218	0.001	0.217
		β	0.000	0.013	0.002	0.003	0.000
		*p*-value	0.079	0.021	0.071	0.000	0.512
Faith’s phylogenetic diversity	Model 1	R2	0.237	0.241	0.237	0.243	0.236
		R2 adj.	0.232	0.236	0.232	0.239	0.231
		β	0.004	0.249	0.030	0.046	0.000
		*p*-value	0.235	0.004	0.141	0.000	0.727
	Model 2	R2	0.253	0.255	0.251	0.257	0.251
		R2 adj.	0.246	0.248	0.244	0.250	0.244
		β	0.005	0.236	0.025	0.044	0.001
		*p*-value	0.139	0.004	0.197	0.000	0.707
Simpson index	Model 1	R2	0.098	0.098	0.098	0.102	0.097
		R2 adj.	0.093	0.092	0.093	0.097	0.091
		β	0.000	0.001	0.000	0.000	0.000
		*p*-value	0.660	0.244	0.085	0.006	0.832
	Model 2	R2	0.104	0.103	0.103	0.106	0.102
		R2 adj.	0.095	0.095	0.095	0.098	0.094
		β	0.000	0.000	0.000	0.000	0.000
		*p*-value	0.719	0.322	0.119	0.018	0.835
Inverse Simpson index	Model 1	R2	0.180	0.180	0.179	0.184	0.177
		R2 adj.	0.175	0.175	0.174	0.179	0.172
		β	0.017	0.457	0.107	0.106	0.002
		*p*-value	0.039	0.026	0.026	0.000	0.517
	Model 2	R2	0.188	0.187	0.186	0.191	0.185
		R2 adj.	0.181	0.179	0.179	0.184	0.177
		β	0.017	0.364	0.093	0.101	0.002
		*p*-value	0.030	0.062	0.040	0.001	0.561

CC, calf circumference; CK, creatinine kinase; TC, thigh circumference.

**Table 4:** The PCR cycle steps, durations and temperatures of the runs.

PCR Cycle Step	Temperature (°C)	Time (min:sec)
Initial denaturation	94	47:00
Denaturation	94	00:47
Annealing	52	00:60
Elongation	72	01:30
Final elongation	72	10:00

°C, Celsius. PCR, polymerase chain reaction; min:sec, minutes:seconds.

## Data Availability

The HELIUS data are owned by the Amsterdam University Medical Centers, located at the AMC in Amsterdam, The Netherlands. Any researcher can request the data by submitting a proposal to the HELIUS Executive Board as outlined at http://www.heliusstudy.nl/en/researchers/collaboration, accessed on 5 December 2021, by email: heliuscoordinator@amsterdamumc.nl. The HELIUS Executive Board will check proposals for compatibility with the general objectives, ethical approval and informed consent forms of the HELIUS study. There are no other restrictions to obtaining the data and all data requests will be processed in the same manner.

## Supplementary Materials

The following are available online at https://www.mdpi.com/article/10.3390/metabo11120858/s1, Figure S1: Food group taxonomy, Figure S2: Comparisons of food groups between the sedentary and active participants, Figure S3: The machine learning of the population without participants with antibiotic use stratified by PA based on the objective method, Table S1: Beta diversity results, Table S2: Comparisons of microbial taxa between the sedentary and active participants, Table S3: Correlations of physical activity related parameters with microbial taxa, Table S4: Comparisons of microbial pathways between the sedentary and active groups, Table S5: Correlations of microbial metabolic pathway with physical activity related parameters, Table S6: Correlations of microbial taxa with food groups.

## References

[1] Owen N., Healy G.N., Dempsey P.C., Salmon J., Timperio A., Clark B.K., Goode A.D., Koorts H., Ridgers N.D., Hadgraft N.T. (2020). Sedentary Behavior and Public Health: Integrating the Evidence and Identifying Potential Solutions. Annu. Rev. Public Health.

[2] Guthold R., Stevens G.A., Riley L.M., Bull F.C. (2018). Worldwide trends in insufficient physical activity from 2001 to 2016: A pooled analysis of 358 population-based surveys with 1·9 million participants. Lancet Glob. Health.

[3] World Health Organization (2018). Global Action Plan on Physical Activity 2018–2030: More Active People for a Healthier World.

[4] Young D.R., Hivert M.-F., Alhassan S., Camhi S.M., Ferguson J.F., Katzmarzyk P.T., Lewis C.E., Owen N., Perry C.K., Siddique J. (2016). Sedentary Behavior and Cardiovascular Morbidity and Mortality: A Science Advisory From the American Heart Association. Circulation.

[5] Wilmot E.G., Edwardson C.L., Achana F.A., Davies M.J., Gorely T., Gray L.J., Khunti K., Yates T., Biddle S.J.H. (2012). Sedentary time in adults and the association with diabetes, cardiovascular disease and death: Systematic review and meta-analysis. Diabetologia.

[6] Patterson R., McNamara E., Tainio M., de Sá T.H., Smith A.D., Sharp S.J., Edwards P., Woodcock J., Brage S., Wijndaele K. (2018). Sedentary behaviour and risk of all-cause, cardiovascular and cancer mortality, and incident type 2 diabetes: A systematic review and dose response meta-analysis. Eur. J. Epidemiol..

[7] Ekelund U., Tarp J., Steene-Johannessen J., Hansen B.H., Jefferis B., Fagerland M.W., Whincup P., Diaz K.M., Hooker S.P., Chernofsky A. (2019). Dose-response associations between accelerometry measured physical activity and sedentary time and all cause mortality: Systematic review and harmonised meta-analysis. BMJ.

[8] Lee I.-M., Shiroma E.J., Lobelo F., Puska P., Blair S.N., Katzmarzyk P.T., Lancet Physical Activity Series Working Group (2012). Effect of physical inactivity on major non-communicable diseases worldwide: An analysis of burden of disease and life expectancy. Lancet.

[9] Arnett D.K., Blumenthal R.S., Albert M.A., Buroker A.B., Goldberger Z.D., Hahn E.J., Himmelfarb C.D., Khera A., Lloyd-Jones D., McEvoy J.W. (2019). 2019 ACC/AHA Guideline on the Primary Prevention of Cardiovascular Disease: A Report of the American College of Cardiology/American Heart Association Task Force on Clinical Practice Guidelines. Circulation.

[10] Cosentino F., Grant P.J., Aboyans V., Bailey C.J., Ceriello A., Delgado V., Federici M., Filippatos G., Grobbee D.E., Hansen T.B. (2020). 2019 ESC Guidelines on diabetes, pre-diabetes, and cardiovascular diseases developed in collaboration with the EASD. Eur. Heart J..

[11] Yumuk V., Tsigos C., Fried M., Schindler K., Busetto L., Micic D., Toplak H. (2015). European Guidelines for Obesity Management in Adults. Obes. Facts.

[12] Bäckhed F., Roswall J., Peng Y., Feng Q., Jia H., Kovatcheva-Datchary P., Li Y., Xia Y., Xie H., Zhong H. (2015). Dynamics and Stabilization of the Human Gut Microbiome during the First Year of Life. Cell Host Microbe.

[13] Manor O., Dai C.L., Kornilov S.A., Smith B., Price N.D., Lovejoy J.C., Gibbons S.M., Magis A.T. (2020). Health and disease markers correlate with gut microbiome composition across thousands of people. Nat. Commun..

[14] Rothschild D., Weissbrod O., Barkan E., Kurilshikov A., Korem T., Zeevi D., Costea P.I., Godneva A., Kalka I.N., Bar N. (2018). Environment dominates over host genetics in shaping human gut microbiota. Nature.

[15] Mailing L.J., Allen J.M., Buford T.W., Fields C.J., Woods J.A. (2019). Exercise and the Gut Microbiome: A Review of the Evidence, Potential Mechanisms, and Implications for Human Health. Exerc. Sport Sci. Rev..

[16] David L.A., Maurice C.F., Carmody R.N., Gootenberg D.B., Button J.E., Wolfe B.E., Ling A.V., Devlin A.S., Varma Y., Fischbach M.A. (2014). Diet rapidly and reproducibly alters the human gut microbiome. Nature.

[17] Johnson A.J., Vangay P., Al-Ghalith G.A., Hillmann B.M., Ward T.L., Shields-Cutler R.R., Kim A.D., Shmagel A.K., Syed A.N., Walter J. (2019). Daily Sampling Reveals Personalized Diet-Microbiome Associations in Humans. Cell Host Microbe.

[18] Barton W., Penney N.C., Cronin O., Garcia-Perez I., Molloy M.G., Holmes E., Shanahan F., Cotter P.D., O’Sullivan O. (2018). The microbiome of professional athletes differs from that of more sedentary subjects in composition and particularly at the functional metabolic level. Gut.

[19] Grosicki G.J., Durk R.P., Bagley J.R. (2019). Rapid gut microbiome changes in a world-class ultramarathon runner. Physiol. Rep..

[20] Munukka E., Ahtiainen J.P., Puigbó P., Jalkanen S., Pahkala K., Keskitalo A., Kujala U.M., Pietilä S., Hollmén M., Elo L. (2018). Six-week endurance exercise alters gut metagenome that is not reflected in systemic metabolism in over-weight women. Front. Microbiol..

[21] Scheiman J., Luber J.M., Chavkin T.A., MacDonald T., Tung A., Pham L.-D., Wibowo M.C., Wurth R.C., Punthambaker S., Tierney B.T. (2019). Meta-omics analysis of elite athletes identifies a performance-enhancing microbe that functions via lactate metabolism. Nat. Med..

[22] Clarke S.F., Murphy E.F., O’Sullivan O., Lucey A.J., Humphreys M., Hogan A., Hayes P., O’Reilly M., Jeffery I.B., Wood-Martin R. (2014). Exercise and associated dietary extremes impact on gut microbial diversity. Gut.

[23] Liu Y., Wang Y., Ni Y., Cheung C.K.Y., Lam K.S.L., Wang Y., Xia Z., Ye D., Guo J., Tse M.A. (2020). Gut Microbiome Fermentation Determines the Efficacy of Exercise for Diabetes Prevention. Cell Metab..

[24] Motiani K.K., Collado M.C., Eskelinen J.-J., Virtanen K.A., Löyttyniemi E., Salminen S., Nuutila P., Kalliokoski K.K., Hannukainen J.C. (2020). Exercise Training Modulates Gut Microbiota Profile and Improves Endotoxemia. Med. Sci. Sports. Exerc..

[25] Clauss M., Gérard P., Mosca A., Leclerc M. (2021). Interplay Between Exercise and Gut Microbiome in the Context of Human Health and Performance. Front. Nutr..

[26] Lampinen E.-K., Eloranta A.-M., Haapala E.A., Lindi V., Väistö J., Lintu N., Karjalainen P., Kukkonen-Harjula K., Laaksonen D., Lakka T.A. (2017). Physical activity, sedentary behaviour, and socioeconomic status among Finnish girls and boys aged 6–8 years. Eur. J. Sport Sci..

[27] Alley S.J., Schoeppe S., Rebar A.L., Hayman M., Vandelanotte C. (2018). Age differences in physical activity intentions and implementation intention preferences. J. Behav. Med..

[28] Loyen A., Nicolaou M., Snijder M.B., Peters R.J.G., Stronks K., Langøien L.J., van der Ploeg H.P., Brug J., Lakerveld J. (2017). Objectively measured sedentary time among five ethnic groups in Amsterdam: The HELIUS study. PLoS ONE.

[29] Sturkenboom S.M., Dekker L.H., Lamkaddem M., Schaap L.A., de Vries J.H., Stronks K., Nicolaou M. (2016). Acculturation and dietary patterns among residents of Surinamese origin in The Netherlands: The HELIUS dietary pattern study. Public Health Nutr..

[30] Deschasaux M., Bouter K.E., Prodan A., Levin E., Groen A.K., Herrema H., Tremaroli V., Bakker G.J., Attaye I., Pinto-Sietsma S.-J. (2018). Depicting the composition of gut microbiota in a population with varied ethnic origins but shared geography. Nat. Med..

[31] Vaccaro J.A., Huffman F.G. (2017). Sex and Race/Ethnicity Differences in Following Dietary and Exercise Recommendations for U.S. Representative Sample of Adults With Type 2 Diabetes. Am. J. Mens Health.

[32] Snijder M.B., Galenkamp H., Prins M., Derks E.M., Peters R.J.G., Zwinderman A.H., Stronks K. (2017). Cohort profile: The Healthy Life in an Urban Setting (HELIUS) study in Amsterdam, The Netherlands. BMJ Open.

[33] Xu K.-Y., Xia G.-H., Lu J.-Q., Chen M.-X., Zhen X., Wang S., You C., Nie J., Zhou H.-W., Yin J. (2017). Impaired renal function and dysbiosis of gut microbiota contribute to increased trimethylamine-N-oxide in chronic kidney disease patients. Sci. Rep..

[34] Bressa C., Bailén-Andrino M., Pérez-Santiago J., González-Soltero R., Pérez M., Montalvo-Lominchar M.G., Maté-Muñoz J.L., Domínguez R., Moreno D., Larrosa M. (2017). Differences in gut microbiota profile between women with active lifestyle and sedentary women. PLoS ONE.

[35] Whisner C.M., Maldonado J., Dente B., Krajmalnik-Brown R., Bruening M. (2018). Diet, physical activity and screen time but not body mass index are associated with the gut microbiome of a diverse cohort of college students living in university housing: A cross-sectional study. BMC Microbiol..

[36] Aya V., Flórez A., Perez L., Ramírez J.D. (2021). Association between physical activity and changes in intestinal microbiota composition: A systematic review. PLoS ONE.

[37] Estaki M., Pither J., Baumeister P., Little J.P., Gill S.K., Ghosh S., Ahmadi-Vand Z., Marsden K.R., Gibson D.L. (2016). Cardiorespiratory fitness as a predictor of intestinal microbial diversity and distinct metagenomic functions. Microbiome.

[38] Allen J.M., Mailing L.J., Niemiro G.M., Moore R., Cook M.D., White B.A., Holscher H.D., Woods J.A. (2018). Exercise Alters Gut Microbiota Composition and Function in Lean and Obese Humans. Med. Sci. Sports Exerc..

[39] Koopen A.M., de Clercq N.C., Warmbrunn M.V., Herrema H., Davids M., de Groot P.F., Kootte R.S., Bouter K.E.C., Nieuwdorp M., Groen A.K. (2020). Plasma Metabolites Related to Peripheral and Hepatic Insulin Sensitivity Are Not Directly Linked to Gut Microbiota Composition. Nutrients.

[40] Nay K., Jollet M., Goustard B., Baati N., Vernus B., Pontones M., Lefeuvre-Orfila L., Bendavid C., Rué O., Mariadassou M. (2019). Gut bacteria are critical for optimal muscle function: A potential link with glucose homeostasis. Am. J. Physiol. Endocrinol. Metab..

[41] van der Kooi A.-L.L.F., Snijder M.B., Peters R.J.G., van Valkengoed I.G.M. (2015). The Association of Handgrip Strength and Type 2 Diabetes Mellitus in Six Ethnic Groups: An Analysis of the HELIUS Study. PLoS ONE.

[42] Studenski S., Perera S., Patel K., Rosano C., Faulkner K., Inzitari M., Brach J., Chandler J., Cawthon P., Connor E.B. (2011). Gait speed and survival in older adults. JAMA.

[43] Weng C.-H., Tien C.-P., Li C.-I., L’Heureux A., Liu C.-S., Lin C.-H., Lin C.-C., Lai S.-W., Lai M.-M., Lin W.-Y. (2018). Mid-upper arm circumference, calf circumference and mortality in Chinese long-term care facility residents: A prospective cohort study. BMJ Open.

[44] Claesson M.J., Jeffery I.B., Conde S., Power S.E., O’Connor E.M., Cusack S., Harris H.M.B., Coakley M., Lakshminarayanan B., O’Sullivan O. (2012). Gut microbiota composition correlates with diet and health in the elderly. Nature.

[45] Fielding R.A., Reeves A.R., Jasuja R., Liu C., Barrett B.B., Lustgarten M.S. (2019). Muscle strength is increased in mice that are colonized with microbiota from high-functioning older adults. Exp. Gerontol..

[46] Zhernakova A., Kurilshikov A., Bonder M.J., Tigchelaar E.F., Schirmer M., Vatanen T., Mujagic Z., Vila A.V., Falony G., Vieira-Silva S. (2016). Population-based metagenomics analysis reveals markers for gut microbiome composition and diversity. Science.

[47] van Etten S., Crielaard L., Muilwijk M., van Valkengoed I., Snijder M.B., Stronks K., Nicolaou M. (2020). Lifestyle clusters related to type 2 diabetes and diabetes risk in a multi-ethnic population: The HELIUS study. Prev. Med..

[48] Jang L.-G., Choi G., Kim S.-W., Kim B.-Y., Lee S., Park H. (2019). The combination of sport and sport-specific diet is associated with characteristics of gut microbiota: An observational study. J. Int. Soc. Sports Nutr..

[49] Röytiö H., Mokkala K., Vahlberg T., Laitinen K. (2017). Dietary intake of fat and fibre according to reference values relates to higher gut microbiota richness in overweight pregnant women. Br. J. Nutr..

[50] Sonnenburg E.D., Smits S.A., Tikhonov M., Higginbottom S.K., Wingreen N.S., Sonnenburg J.L. (2016). Diet-induced extinctions in the gut microbiota compound over generations. Nature.

[51] Ma W., Nguyen L.H., Song M., Wang D.D., Franzosa E.A., Cao Y., Joshi A., Drew D.A., Mehta R., Ivey K.L. (2021). Dietary fiber intake, the gut microbiome, and chronic systemic inflammation in a cohort of adult men. Genome Med..

[52] Deehan E.C., Yang C., Perez-Muñoz M.E., Nguyen N.K., Cheng C.C., Triador L., Zhang Z., Bakal J.A., Walter J. (2020). Precision Microbiome Modulation with Discrete Dietary Fiber Structures Directs Short-Chain Fatty Acid Production. Cell Host Microbe.

[53] Verhaar B.J.H., Collard D., Prodan A., Levels J.H.M., Zwinderman A.H., Bäckhed F., Vogt L., Peters M.J.L., Muller M., Nieuwdorp M. (2020). Associations between gut microbiota, faecal short-chain fatty acids, and blood pressure across ethnic groups: The HELIUS study. Eur. Heart J..

[54] Bui T.P.N., Mannerås-Holm L., Puschmann R., Wu H., Troise A.D., Nijsse B., Boeren S., Bäckhed F., Fiedler D., DeVos W.M. (2021). Conversion of dietary inositol into propionate and acetate by commensal Anaerostipes associates with host health. Nat. Commun..

[55] Cicero N., Albergamo A., Salvo A., Bua G.D., Bartolomeo G., Mangano V., Rotondo A., Di Stefano V., Di Bella G., Dugo G. (2018). Chemical characterization of a variety of cold-pressed gourmet oils available on the Brazilian market. Food Res. Int..

[56] Koistinen V.M., Mattila O., Katina K., Poutanen K., Aura A.-M., Hanhineva K. (2018). Metabolic profiling of sourdough fermented wheat and rye bread. Sci. Rep..

[57] Bento-Silva A., Koistinen V.M., Mena P., Bronze M.R., Hanhineva K., Sahlstrøm S., Kitrytė V., Moco S., Aura A.-M. (2019). Factors affecting intake, metabolism and health benefits of phenolic acids: Do we understand individual variability?. Eur. J. Nutr..

[58] Mokkala K., Houttu N., Cansev T., Laitinen K. (2020). Interactions of dietary fat with the gut microbiota: Evaluation of mechanisms and metabolic consequences. Clin. Nutr..

[59] Abu-Ghazaleh N., Chua W.J., Gopalan V. (2021). Intestinal microbiota and its association with colon cancer and red/processed meat consumption. J. Gastroenterol. Hepatol..

[60] Bel Lassen P., Attaye I., Adriouch S., Nicolaou M., Aron-Wisnewsky J., Nielsen T., Chakaroun R., Le Chatelier E., Forslund S., Belda E. (2021). Protein Intake, Metabolic Status and the Gut Microbiota in Different Ethnicities: Results from Two Independent Cohorts. Nutrients.

[61] Vujkovic-Cvijin I., Sklar J., Jiang L., Natarajan L., Knight R., Belkaid Y. (2020). Host variables confound gut microbiota studies of human disease. Nature.

[62] Nicolaou M., Gademan M.G.J., Snijder M.B., Engelbert R.H.H., Dijkshoorn H., Terwee C.B., Stronks K. (2016). Validation of the SQUASH Physical Activity Questionnaire in a Multi-Ethnic Population: The HELIUS Study. PLoS ONE.

[63] Laudadio I., Fulci V., Palone F., Stronati L., Cucchiara S., Carissimi C. (2018). Quantitative Assessment of Shotgun Metagenomics and 16S rDNA Amplicon Sequencing in the Study of Human Gut Microbiome. OMICS.

[64] Durazzi F., Sala C., Castellani G., Manfreda G., Remondini D., De Cesare A. (2021). Comparison between 16S rRNA and shotgun sequencing data for the taxonomic characterization of the gut microbiota. Sci. Rep..

[65] Newland G.A.I., Gibson G.R., Jackson F.L., Wijeyesekera A. (2021). Assessment of stool collection and storage conditions for in vitro human gut model studies. J. Microbiol. Methods.

[66] Wendel-Vos S., van den Berg M., Duijvestijn M., de Hollander E. (2019). Beweegrichtlijnen en Wekelijks Sporter. RIVM-Briefrapport 2019-0237.

[67] Bull F.C., Al-Ansari S.S., Biddle S., Borodulin K., Buman M.P., Cardon G., Carty C., Chaput J.-P., Chastin S., Chou R. (2020). World Health Organization 2020 guidelines on physical activity and sedentary behaviour. Br. J. Sports Med..

[68] Brage S., Brage N., Franks P.W., Ekelund U., Wareham N.J. (2005). Reliability and validity of the combined heart rate and movement sensor Actiheart. Eur. J. Clin. Nutr..

[69] Beukers M.H., Dekker L.H., de Boer E.J., Perenboom C.W.M., Meijboom S., Nicolaou M., de Vries J.H.M., Brants H.A.M. (2015). Development of the HELIUS food frequency questionnaires: Ethnic-specific questionnaires to assess the diet of a multiethnic population in The Netherlands. Eur. J. Clin. Nutr..

[70] Edgar R.C. (2010). Search and clustering orders of magnitude faster than BLAST. Bioinformatics.

[71] Quast C., Pruesse E., Yilmaz P., Gerken J., Schweer T., Yarza P., Peplies J., Glöckner F.O. (2013). The SILVA ribosomal RNA gene database project: Improved data processing and web-based tools. Nucleic Acids Res..

[72] Callahan B.J., McMurdie P.J., Rosen M.J., Han A.W., Johnson A.J.A., Holmes S.P. (2016). DADA2: High-resolution sample inference from Illumina amplicon data. Nat. Methods.

[73] Katoh K., Standley D.M. (2013). MAFFT multiple sequence alignment software version 7: Improvements in performance and usability. Mol. Biol. Evol..

[74] Katoh K., Misawa K., Kuma K., Miyata T. (2002). MAFFT: A novel method for rapid multiple sequence alignment based on fast Fourier transform. Nucleic Acids Res..

[75] Price M.N., Dehal P.S., Arkin A.P. (2010). FastTree 2—Approximately maximum-likelihood trees for large alignments. PLoS ONE.

[76] McMurdie P.J., Holmes S. (2013). phyloseq: An R package for reproducible interactive analysis and graphics of microbiome census data. PLoS ONE.

[77] Lozupone C., Knight R. (2005). UniFrac: A new phylogenetic method for comparing microbial communities. Appl. Environ. Microbiol..

[78] Benjamini Y., Drai D., Elmer G., Kafkafi N., Golani I. (2001). Controlling the false discovery rate in behavior genetics research. Behav. Brain Res..

