# Supplementary material for: Physical Activity and Dietary Composition Relate to Differences in Gut Microbial Patterns in a Multi-Ethnic Cohort—The HELIUS Study
Source: Metabolites. 2021 Dec 9;11(12):858. doi: 10.3390/metabo11120858 (PMC8707449; doi:10.3390/metabo11120858)
Supplement: Supplementary file 1 [file metabolites-11-00858-s001.zip › metabolites-1446002-SI/metabolites_1446002_Suppl 2.0/MDPI_metabolites_Supplementary_Figure_S1-S3.pdf]

Supplementary Figure S1: Food group taxonomy of food groups derived from ethnic-specific food frequency questionnaire (FFQ).

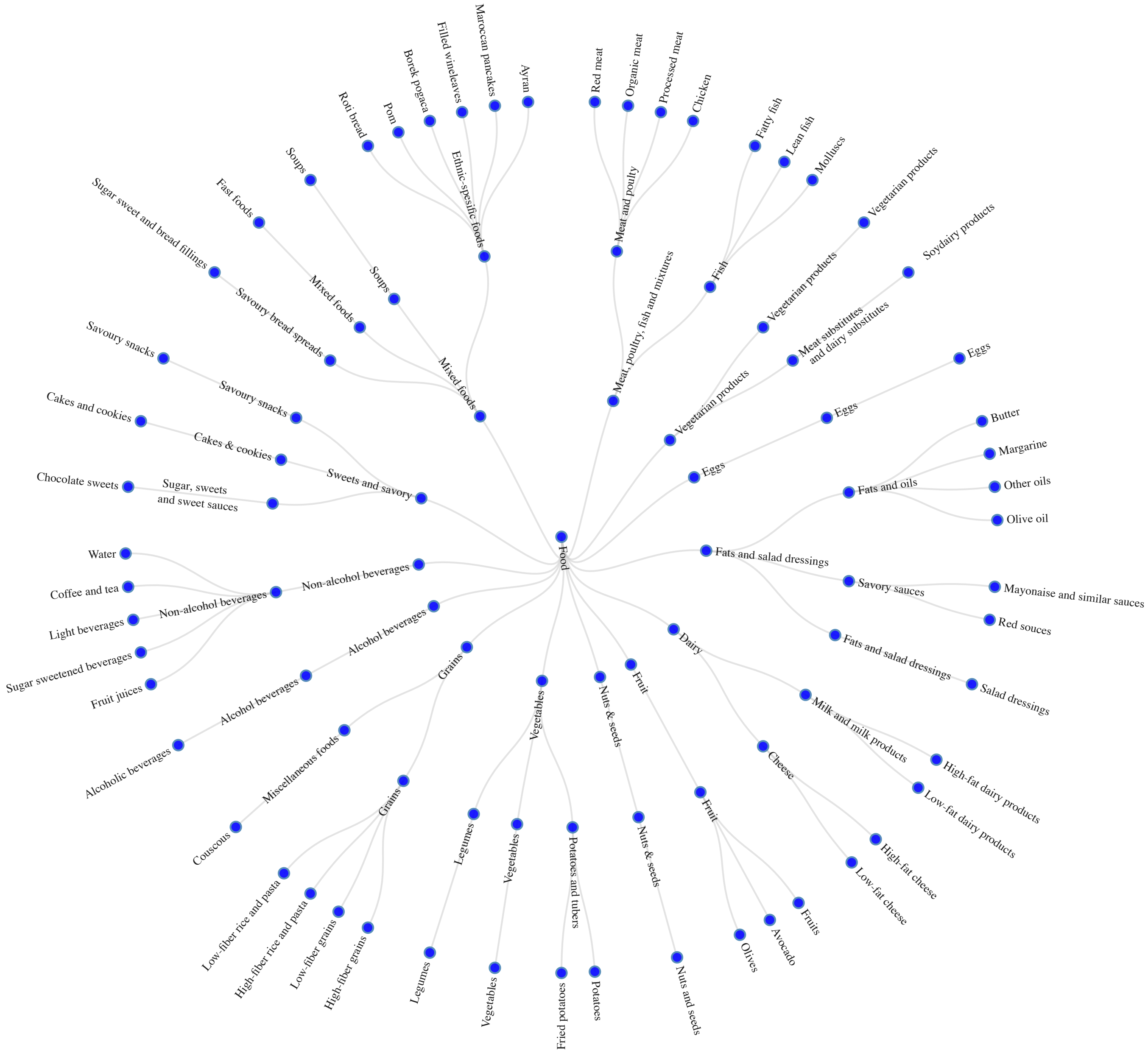

Supplementary Figure S2 (*Cont.*): Comparisons of food groups between the sedentary and active groups. Wilcoxon test was used as statistical test, p-values were corrected with Benjamini-Hochberg. Red dots, Sedentary; Blue dots, active.

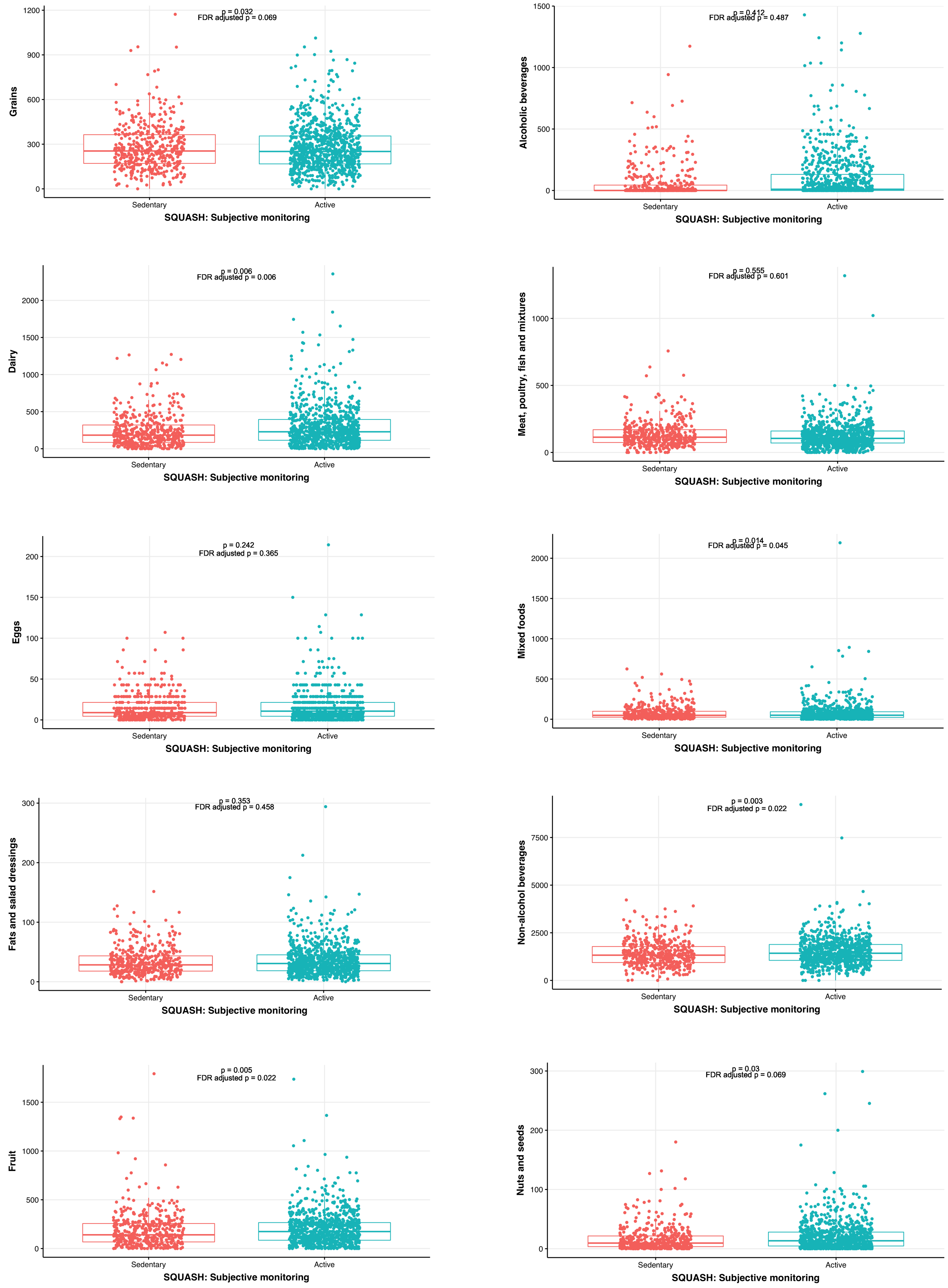

Supplementary Figure S2: Comparisons of food groups between the sedentary and active groups. Wilcoxon test was used as statistical test, p-values were corrected with Benjamini-Hochberg. Red dots, Sedentary; Blue dots, active.

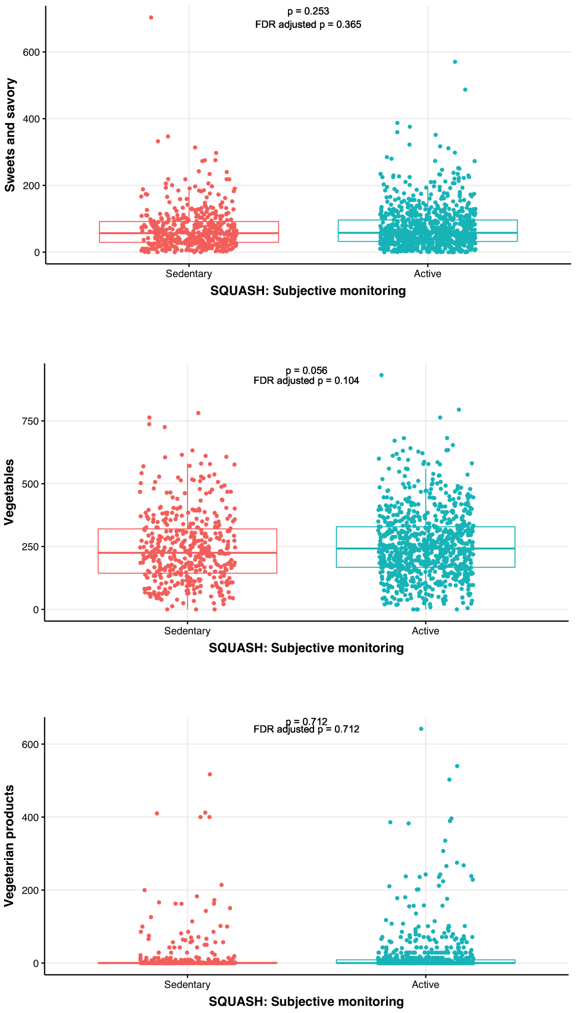

Supplementary Figure S3: The relative variable importance for the 15 most important amplicon sequence variants (ASVs), annotated with the highest taxonomic classification from the machine learning of the population without antibiotic use stratified by PA based on the objective method (ActiHeart).

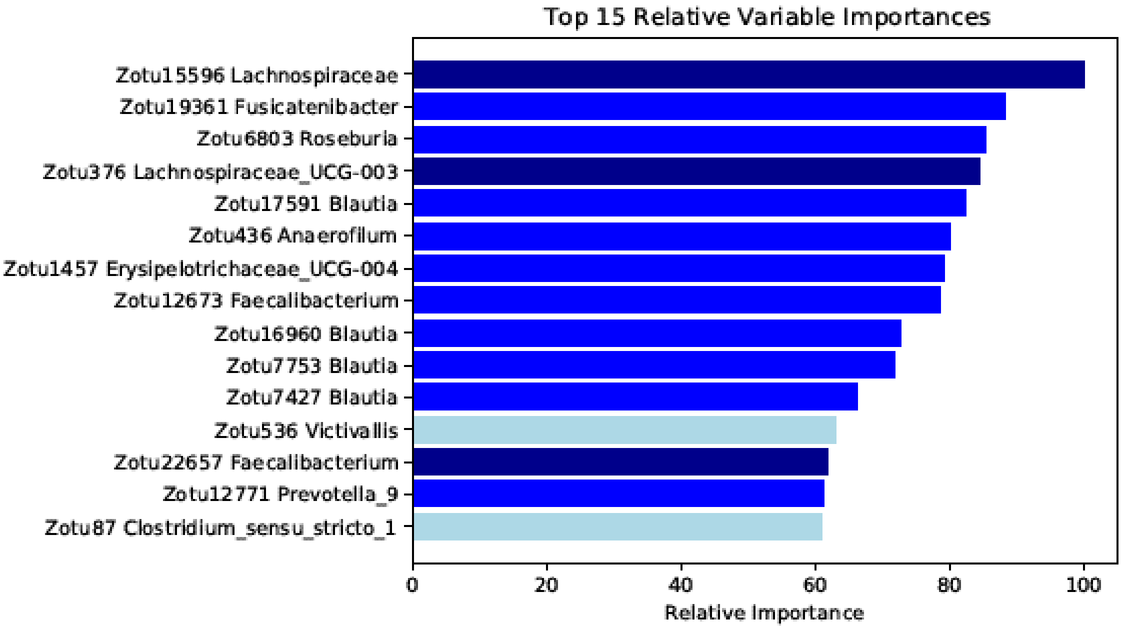


AUC 0.74 ± 0.08, n=151
